# Enhanced Human T Lymphocyte Antigen Priming by Cytokine-Matured Dendritic Cells Overexpressing Bcl-2 and IL-12

**DOI:** 10.3389/fcell.2020.00205

**Published:** 2020-03-27

**Authors:** Hui Zhang, Yu Wang, Qian-Ting Wang, Sheng-Nan Sun, Shi-You Li, Hong Shang, You-Wen He

**Affiliations:** ^1^NHC Key Laboratory of AIDS Immunology (China Medical University), Department of Laboratory Medicine, The First Affiliated Hospital of China Medical University, Shenyang, China; ^2^National Clinical Research Center for Laboratory Medicine, The First Affiliated Hospital of China Medical University, Shenyang, China; ^3^Life Science Institute, Jinzhou Medical University, Jinzhou, China; ^4^Beijing Tricision Biotherapeutics Inc., Beijing, China; ^5^Department of Immunology, Duke University Medical Center, Durham, NC, United States

**Keywords:** dendritic cell, T cell responses, survival, Bcl-2, IL-12p70

## Abstract

Dendritic cell (DC)-based vaccination is a promising immunotherapeutic strategy for cancer. However, clinical trials have shown only limited efficacy, suggesting the need to optimize protocols for human DC vaccine preparation. In this study, we systemically compared five different human DC vaccine maturation protocols used in clinical trials: (1) a four-cytokine cocktail (TNF-α, IL-6, IL-1β, and PGE2); (2) an α-DC-cytokine cocktail (TNF-α, IL-1β, IFN-α, IFN-γ, and poly I:C); (3) lipopolysaccharide (LPS)/IFN-γ; (4) TNF-α and PGE2; and (5) TriMix (mRNAs encoding CD40L, CD70, and constitutively active Toll-like receptor 4 electroporated into immature DCs). We found that the four-cytokine cocktail induced high levels of costimulatory and HLA molecules, as well as CCR7, in DCs. Mature DCs (mDCs) matured with the four-cytokine cocktail had higher viability than those obtained with the other protocols. Based on these features, we chose the four-cytokine cocktail protocol to further improve the immunizing capability of DCs by introducing exogenous genes. We showed that introducing exogenous Bcl-2 increased DC survival. Furthermore, introducing IL-12p70 rescued the inhibition of IL-12 secretion by PGE2 without impairing the DC phenotype. Introducing both Bcl-2 and IL-12p70 mRNAs into DCs induced enhanced cytomegalovirus pp65-specific CD8^+^ T cells secreting IFN-γ and TNF-α. Taken together, our data suggest that DC matured by the four-cytokine cocktail combined with exogenous Bcl-2 and IL-12p70 gene expression represents a promising approach for clinical applications in cancer immunotherapy.

## Introduction

Dendritic cell (DC)-based vaccination can be used to induce host antitumor immunity and has shown promising clinical efficacy against some tumors (Yu et al., 2004; De Vleeschouwer et al., 2008; Cho et al., 2012; Mitchell et al., 2015; Erhart et al., 2018; Reap et al., 2018). Nevertheless, most clinical trials using DC vaccines, prepared with a variety of protocols, in cancer therapy show only limited efficacy, suggesting the need to optimize these clinical protocols (Vandenberk et al., 2015; Constantino et al., 2016). Effective induction of antitumor T cell responses requires clinical-grade DC vaccines that possess the following features: expression of high levels of costimulatory molecules (Dhodapkar et al., 2001); the ability to migrate toward T cell areas in the lymph nodes (De Vries et al., 2003); and secretion of cytokines to prime immune responses (Steinman et al., 2003; Hunter, 2005). Thus, a protocol to prepare a human DC vaccine with the above characteristics is desirable for clinical applications.

Currently, at least five different human DC vaccine maturation protocols have been used in clinical trials. These protocols include a four-cytokine cocktail (TNF-α, IL-6, IL-1β, and PGE2) (Lee et al., 2002; Scandella et al., 2004; Batich et al., 2017); an α-DC cytokine cocktail (TNF-α, IL-1β, IFN-α, IFN-γ, and poly I:C) (Mailliard et al., 2004; Lee et al., 2008; Park et al., 2011; Akiyama et al., 2012); LPS plus IFN-γ (Dohnal et al., 2007), TNF-α plus PGE2 (Holtl et al., 1999); and TriMix DC (electroporation of a mixture of CD40L, CD70, and a constitutively active form of Toll-like receptor 4 (caTLR4) mRNAs into immature DCs) (Van Nuffel et al., 2012; Benteyn et al., 2013). These protocols use combinations of DC maturation signals, such as pathogen-associated molecular patterns (LPS and poly I:C), T cell-dependent signals (IFN-γ and CD40L), and inflammatory cytokines (TNF-α, IL-6, and IL-1β), to upregulate the expression of costimulatory molecules, HLA, and chemokine receptors. This enables the resulting DC vaccines to present antitumor antigens and prime T cells.

Although the above protocols have been tested in clinical trials, their capacities to induce functional mDCs have not been systematically compared. These protocols are likely to have distinct capabilities to modulate human DC function. For example, the four-cytokine cocktail has been demonstrated to induce the upregulation of DC maturation markers but no IL-12p70 (Lee et al., 2002). The α-DC cytokine cocktail matured DCs can produce high level of IL-12p70, but show lower efficiency to express exogenous mRNA genes (Bontkes et al., 2007). TLR agonists, such as poly I:C, TLR4 agonists, and LPS stimulated DCs produce high level of proinflammatory cytokines and tumor specific T cell responses (Bogunovic et al., 2011). TriMix DC has been reported to induce a lower number of epitopes specific T cells (Bonehill et al., 2009). In this study, we systemically compared the five clinical DC maturation protocols with respect to their overall capability to generate potent human DC vaccines. Based on this comparison, we chose the four-cytokine cocktail protocol for further improvement of T cell priming capability by introducing the anti-apoptotic gene Bcl-2 and the antitumor T cell priming cytokine IL-12. Our results show that the four-cytokine cocktail combined with electroporation of Bcl-2 and IL-12p70 mRNAs represents a promising approach for producing DC vaccines.

## Materials and Methods

### Cytokines and Antibodies

Monoclonal antibodies [PE-anti-hCD40 (clone 5C3, 334308), PE-anti-hCD80 (clone 2D10, 305208), PE-anti-hCD83 (clone HB15e, 305308), PE-anti-hCD86 (clone BU63, 374206), PE-anti-hHLA-ABC (clone W6/32, 311406), PE-anti-hHLA-DR (clone L243, 307606), PE-anti-hCCR7 (clone G043H7, 353204), Alexia Fluor^®^ 647-anti-hBcl-2 (clone 100, 658706), APC-anti-hIFN-γ (clone 4S.B3, 502512), Percp-cy5.5-anti-hTNF-α (clone MAb11, 502926) and APC-cy7-anti-hCD8 (clone SK1, 344722) and cytokines (human granulocyte-macrophage colony-stimulating factor (GM-CSF) (572902), IL-4 (574002), IL-6 (570802), IL-1β (579402), TNF-α (570102), and IFN-γ (570202)] were obtained from BioLegend. Poly I:C (P9582), LPS (L2630), and PGE2 (P5640) were purchased from Sigma. IFN-α (130-093-873) was purchased from Miltenyi Biotec Inc.

### mRNA

The pSP73-CD40L-Sph/A64, pSP73-caTLR4-Sph/A64, and pSP73-cytomegalovirus (CMV) pp65-Sph/A64 plasmids were as described previously (Nair et al., 2014). Total RNA was extracted from human peripheral blood mononuclear cells (PBMCs) and reverse-transcribed using oligo dT primers. Bcl-2 cDNA was PCR-amplified with primers containing *Pac*I and *Xma*I restriction sites. The forward primer was 5′-CAGTTAATTAATCACTTGTGGCCCAGATAGGCACCC-3′ and the reverse primer was 5′-TTAACCCGGGTCACTTGTGGC CCAGATAGGCACCC-3′. CD70 was amplified using the forward primer 5′-AACTTCTAGAGCCACCATGCCGGAGGA GGGTTC-3′ and the reverse primer 5′-TTAACCCGGGTC AGGGGCGCACCCAC-3′. IL-12p70 was amplified from the pUNO1-hIL-12(p40p35) plasmid (InvivoGen, puno1-hil12ba). All the fragments were cloned into the pSP73-Sph/A64 vector, then the plasmids were linearized with *Spe*I and used as templates for RNA synthesis. The *in vitro* transcription was carried out using T7 RNA polymerase (mMESSAGE mMACHINE Kit; Ambion) according to the manufacturer’s instructions. mRNA quality was checked by agarose gel electrophoresis, and the concentration was measured by spectrophotometric analysis. mRNA was stored at −80°C in small aliquots.

### DC Preparation

Peripheral blood mononuclear cells were separated by density gradient centrifugation from 90 ml whole blood samples from healthy donors. Monocytes were obtained by being allowed to adhere to plastic for 2 h in 75 cm^2^ culture flasks. Non-adherent cells were harvested and frozen. Immature DCs and mDCs were induced as follows. Adherent cells were cultured with 15 ml AIM-V media (Invitrogen) containing 800 U/ml GM-CSF and 500 U/ml human IL-4 at 37°C for 6 days (immature DCs) (Nair et al., 2012). On day 7, cells were collected and seeded in 12-well plates at 1 × 10^6^ cells/ml and then activated with different maturation stimuli (mDCs): four-cytokine cocktail (160 ng/ml IL-6, 5 ng/ml IL-1β, 5 ng/ml TNF-α, and 1 μg/ml PGE2) (Lee et al., 2002; Scandella et al., 2004; Batich et al., 2017); α-DC-cytokine cocktail (10 ng/ml IL-1β, 50 ng/ml TNF-α, 3,000 IU/q IFN-α, 1,000 IU/ml IFN-γ, and 20 μg/ml poly I:C) (Mailliard et al., 2004; Lee et al., 2008; Park et al., 2011; Akiyama et al., 2012); 100 ng/ml LPS plus 1,000 IU/ml IFN-γ (Dohnal et al., 2007); and 10 ng/ml TNF-α plus 10 μg/ml PGE2 (Holtl et al., 1999).

### Electroporation of DCs

Electroporation of DCs was performed as previously described (Nair et al., 2014). Briefly, DCs were harvested and washed once with phosphate-buffered saline (PBS) and once with Opti-MEM without phenol red (Invitrogen Life Technologies, 11058021). The cells were resuspended in Opti-MEM (5 × 10^6^ cells/ml). A 200 μl volume of cell suspension and RNA were transferred to a 4 mm cuvette and pulsed using an Electro Square Porator (ECM630, BTX, San Diego, CA, United States). Pulse conditions were: voltage, 300 V; capacitance, 150 μF; and resistance, 25 Ω. A 5 μg volume of mRNA/10^6^ DCs was used for CD40L, caTLR4, CD70, pp65, and Bcl-2 mRNA electroporation. Cells were transferred to medium immediately after electroporation.

### Flow Cytometry Analysis of Maturation Phenotype and Cell Death

Dendritic cells were stained with antibodies against maturation phenotype markers, CCR7 expression and a live/dead dye kit (Invitrogen, L34955) for maturation and viability analysis at room temperature for 20 min, washed twice with PBS + 0.1% fetal bovine serum (FBS) and analyzed by flow cytometry. The DC population was gated according to its forward scatter (FSC) and side scatter (SSC) properties. The expression of CD40, CD80, CD83, CD86, HLA-ABC, HLA-DR, and CCR7 on live DCs was analyzed after 24 h of stimulation. Corresponding isotypes were used as negative control. Data were acquired using a FACS CantoII flow cytometer (BD) and analyzed using FlowJo software.

### Detection of DC Cytokine Secretion

The supernatants from mDCs matured by different stimuli for 24 h were collected for IL-12p70 analysis. For the IL-12p70 mRNA electroporation experiment, mDCs matured with the four-cytokine cocktail were electroporated with different amounts of IL-12p70 mRNAs (0.625, 1.25, 2.5, and 5 μg mRNAs per 10^6^ DCs), and the supernatants were collected after 24 h of electroporation. IL-12p70 was detected by ELISA (eBioscience, 88-7126-88) according to the manufacturer’s instructions.

### In vitro Stimulation of T Cells With mRNA-Loaded DCs

After electroporation with pp65, pp65 + Bcl-2, or pp65 + Bcl-2 + IL-12p70 mRNAs, DCs were rested in DC culture medium at 37°C for the appropriate time. The mRNA-loaded DCs were immediately used for T cell expansion or cryoconserved for T cell re-stimulation. For pre-stimulation, non-adherent cells were thawed, resuspended in PBS, and treated with 200 U/ml DNase I at 37°C for 20 min. Then the cells were stimulated with mRNA-loaded mDCs at a responder-to-stimulator ratio of 10:1. Next, 2 × 10^4^ non-adherent cells were co-cultured with 2 × 10^3^ DCs in 200 μl T cell stimulation medium (RPMI 1640 with 10% FBS, 2 mM L-glutamine, 20 mM HEPES, 1 mM sodium pyruvate, 0.1 mM MEM-non-essential amino acids, 100 IU/ml penicillin, 100 μg/ml streptomycin, and 5 × 10^5^ M β-mercaptoethanol) in a 96-well flat-bottomed plate in the presence of 10 ng/ml IL-7. On day 5, 50 U/ml IL-2 was added. Non-adherent cells were re-stimulated with DCs electroporated with the appropriate mRNAs on day 7. IL-2 and IL-7 were supplied every 3 days. On day 14, non-adherent cells were harvested and used as effector T cells, and mRNA-loaded DCs were used as targets. The pp65-specific CD8^+^ T cell response was determined using intracellular staining of IFN-γ and TNF-α. After 4 h of incubation in the presence of monensin (BioLegend), cells were collected and stained with APC-cy7-anti-hCD8 and live/dead dye, then fixed in 4% paraformaldehyde and permeabilized in 0.1% saponin (BioLegend). After washing, cells were stained with APC-anti-hIFN-γ and percp-cy5.5-anti-hTNF-α. The results were analyzed using FlowJo software.

### Statistical Analysis

Data are presented as mean ± standard error of the mean. Statistical analyses were performed with Prism 6.0 (GraphPad Software Inc., United States), using *t*-tests to compare differences between groups. A *p*-value < 0.05 was considered statistically significant.

## Results

### Induction of DC Maturation by Five Clinical Protocols

**FIGURE 1:**
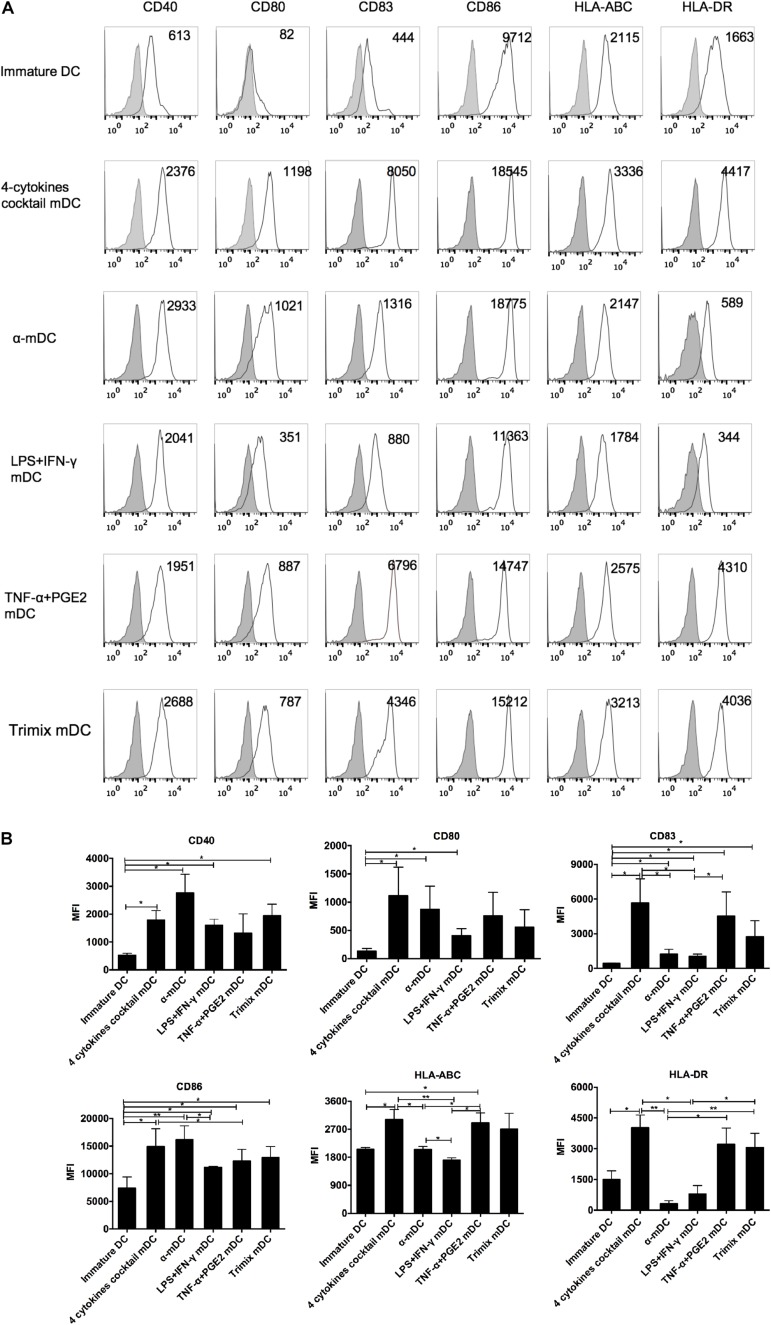
Phenotypic analysis of DCs matured with five clinical protocols. Adherent cells from PBMCs were cultured with GM-CSF and IL-4 for 6 days (immature DCs) and then stimulated with the five maturation protocols for 24 h. **(A)** Expression of maturation-related markers on matured DCs. Numbers in the top right corner represent the mean fluorescence intensity (MFI) of the indicated molecules. Gray shading in histograms represents isotype controls. Data are representative of three independent experiments. **(B)** Summary of the expression of the indicated surface markers, from three independent experiments (*n* = 3). ^∗^*p* ≤ 0.05, ^∗∗^*p* ≤ 0.01.

Phenotypic maturation of DCs is essential for their functional capacity and activation of T cells (Vandenberk et al., 2015; Constantino et al., 2016). Different maturation stimuli may induce DC maturation to various degrees. We compared five different protocols used to produce mDCs in clinical trials: a four-cytokine cocktail (TNF-α, IL-6, IL-1β, and PGE2); a α-DC-cytokine cocktail (TNF-α, IL-1β, IFN-α, IFN-γ, and poly I:C); LPS plus IFN-γ; TNF-α plus PGE2; and TriMix electroporation. As expected, all five treatments had induced DC maturation 24 h post stimulation (Figure 1A). The mDCs induced by the four-cytokine cocktail showed a significantly higher level of all six surface markers (CD40, CD80, CD83, CD86, HLA-ABC, and HLA-DR) compared with those induced by the other protocols (Figure 1B). By comparison, mDCs induced by the α-DC-cytokine cocktail protocol had high expression levels of CD40, CD80, and CD86 but low levels of CD83, and failed to upregulate HLA-ABC and HLA-DR (Figure 1B). Similar to the four-cytokine cocktail protocol, the TNF-α plus PGE2 and TriMix protocols induced upregulation of all six surface markers but at overall lower levels (Figure 1B). These data demonstrate that the five protocols have distinct capabilities to upregulate DC surface co-stimulatory molecules and HLA expression.

### CCR7 Expression on DCs Matured by Five Clinical Protocols

**FIGURE 2:**
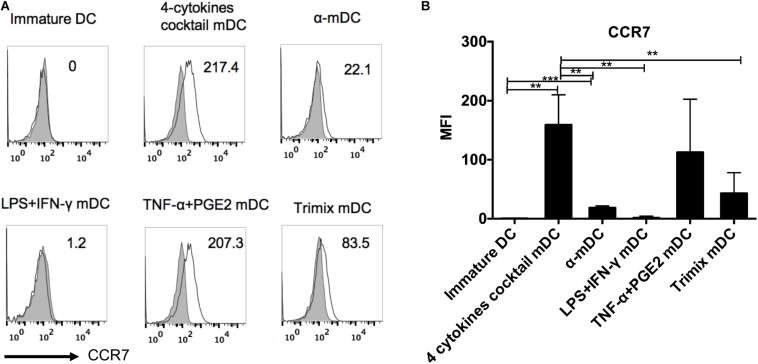
Surface expression of CCR7 on DCs matured with five clinical protocols. Adherent cells from PBMCs were cultured with GM-CSF and IL-4 for 6 days (immature DCs) and then stimulated with the five maturation protocols for 24 h. The expression of CCR7 on live DCs was analyzed. **(A)** Representative histograms of CCR7 expression (black line). Gray shading in histograms represents isotype controls. Numbers in the top right corners represent the MFI of CCR7 expression. **(B)** Summary of MFI of CCR7 expression from three independent experiments (*n* = 3). ^∗∗^*p* ≤ 0.01, ^∗∗∗^*p* ≤ 0.001.

CCR7 expression endows DCs with the ability to migrate to draining lymphoid nodes, which is essential to prime effective T cell responses (Charbonnier et al., 1999; Cyster, 1999; Tal et al., 2011). To compare CCR7 expression levels on DCs upon maturation stimulations, we analyzed CCR7 on DCs matured with the five clinical protocols described above five clinical protocols described above five clinical protocols described above. As shown in Figure 2, the four-cytokine cocktail and TNF-α plus PGE2 protocols both strongly upregulated CCR7 expression, whereas the α-DC-cytokine cocktail and TriMix only slightly induced CCR7 expression on mDCs. The LPS plus IFN-γ maturation protocol failed to induce significant upregulation of CCR7 (Figure 2). Protocols containing PEG2 readily upregulate CCR7, according to several studies (Scandella et al., 2002; Kabashima et al., 2003; Legler et al., 2006).

### Effects of Different Clinical Protocols on DC Survival

**FIGURE 3:**
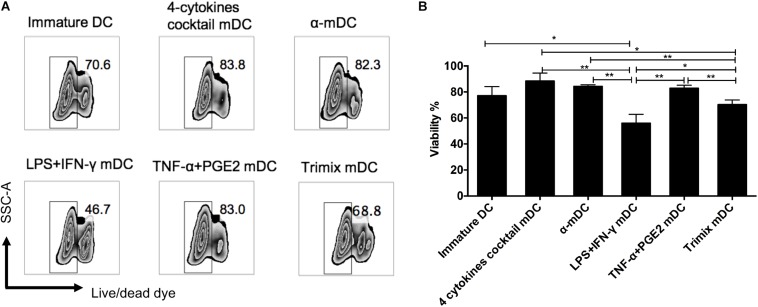
Effects of different maturation protocols on DC survival. DCs matured with five clinical protocols for 24 h were stained with cell viability dye. **(A)** Representative FACS plots of cell survival. **(B)** Comparison of viability of DCs matured with different maturation stimuli. The data represent three independent experiments (*n* = 3). ^∗^*p* ≤ 0.05, ^∗∗^*p* ≤ 0.01.

Prolonged survival of a DC vaccine is likely to enable continuous priming of T cells *in vivo*. We therefore assessed the viability of mDCs 24 h after different maturation stimuli by flow cytometry. The mDCs maintained high levels of viability after maturation by three protocols: the four-cytokine cocktail, the α-DC-cytokine cocktail, and TNF-α plus PGE2 (Figure 3). By contrast, the mDCs displayed reduced viability when matured by the LPS plus IFN-γ or the TriMix protocols (Figure 3).

### Bcl-2 mRNA Transduction Enhances Human DC Survival and DC-Mediated Antigen-Specific CD8^+^ T Cell Responses

**FIGURE 4:**
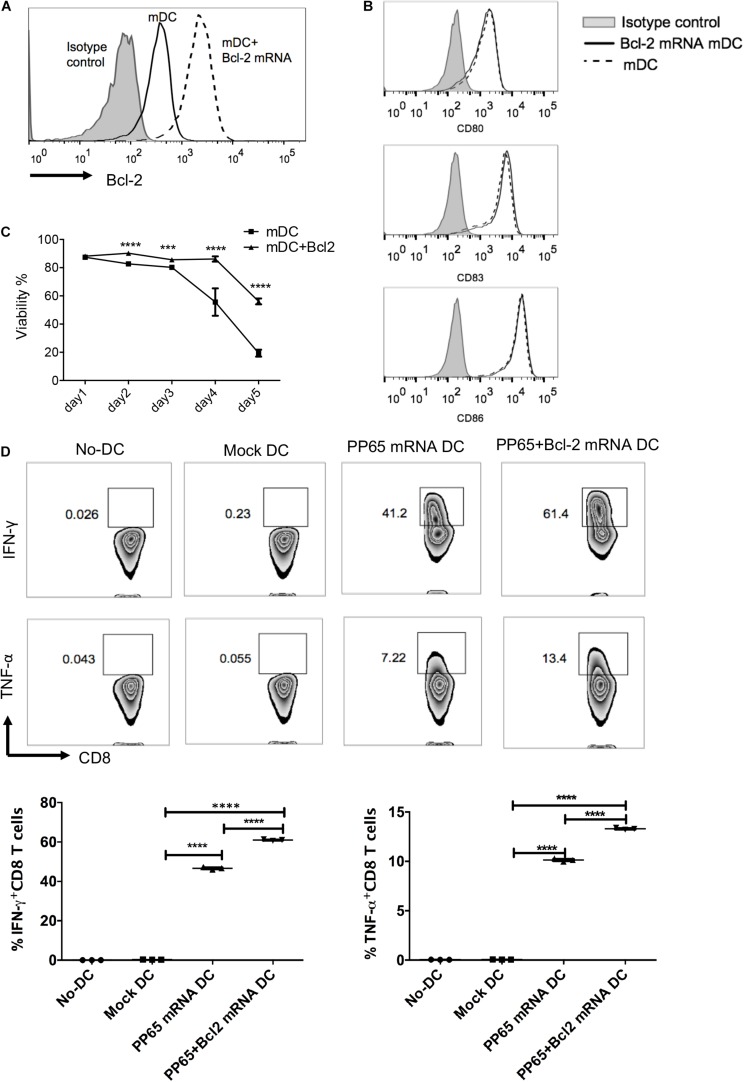
Exogenous Bcl-2 enhances human DC survival and priming of antigen-specific CD8^+^ T cell responses. Immature DCs were matured with the four-cytokine cocktail for 24 h; mDCs were electroporated with Bcl-2 mRNA (5 μg/10^6^ DCs). Expression of Bcl-2 and co-stimulatory molecules and cell viability were evaluated 24 h after transfection. **(A)** Representative histograms of Bcl-2 expression after electroporation. **(B)** Representative histograms of CD80, CD83, and CD86 expression on DCs with and without Bcl-2 transfection. **(C)** Time course analysis of cell survival. Data are representative of three independent experiments. **(D)** Antigen-specific CD8^+^ T cells stimulated with CMV-pp65 loaded DCs. mDCs were electroporated with pp65 or pp65 + Bcl-2 mRNAs. After 4 h, the mRNA-loaded mDCs were washed and co-cultured with non-adherent cells at a 1:10 ratio in the presence of IL-7. IL-2 was added on day 5. The non-adherent cells were re-stimulated with mRNA-loaded DC cells on day 7. On day 14, pp65 spcific-CD8^+^ T cell responses (IFN-γ and TNF-α production) were detected after 4 h of re-stimulation with mRNA-loaded DCs. The no-DC control consisted of non-adherent cells cultured under the same conditions; the mock-DC controls consisted of DC cells without mRNA electroporation. The results of one of three independent experiments are shown (*n* = 3). ^∗∗∗^*p* ≤ 0.001, ^****^p ≤ 0.0001.

The purpose of this study was to optimize current human DC vaccine protocols. On the basis of the upregulation of co-stimulatory molecules and CCR7 and the improvements in cell viability of mDCs prepared by the five protocols, we chose the four-cytokine cocktail protocol for further optimization. A previous study reported rapid downregulation of the anti-apoptotic protein Bcl-2 upon DC maturation (Granucci et al., 2001a). We hypothesized that exogenous Bcl-2 expression would prolong human mDC survival and enhance human CD8^+^ T cell priming. We electroporated Bcl-2 mRNA into DCs matured by the four-cytokine cocktail protocol (Figure 4A). After 24 h, the cytokine cocktail was removed and DC survival was measured. Exogenous Bcl-2 expression did not affect the maturation phenotype of mDCs based on CD80, CD83, and CD86 expression (Figure 4B). As expected, the viability of mDCs was significantly increased after Bcl-2 mRNA electroporation during the 5-day culture period compared with unmanipulated DCs.

To determine whether prolonged DC survival could improve T cell responses, we evaluated the capacity of mDCs expressing exogenous Bcl-2 to prime human CMV-specific CD8^+^ T cells *in vitro*. The mDCs were electroporated with CMV pp65 and pp65 + Bcl-2 mRNAs, respectively and co-cultured with autologous T cells. The cells were re-stimulated on day 7 with the corresponding mRNA-loaded DCs. Antigen-specific CD8^+^ T cells were identified at day 14. The mDCs transduced with pp65 mRNAs induced a high level of T cell responses in three donors. The percentage of IFN-γ^+^ CD8^+^ T cells reached 41% after 2 weeks of expansion (Figure 4D). DCs electroporated with pp65 + Bcl-2 mRNAs could prime significantly elevated antigen-specific effector CD8^+^ T cells as assessed by IFN-γ and TNF-α production (Figure 4D). These results suggest that mDCs expressing exogenous Bcl-2 have an enhanced capability to prime CMV-specific CD8^+^ T cells.

### Combination of IL-12p70 and Bcl-2 mRNA Electroporation Further Enhances DC Priming Capability

**FIGURE 5:**
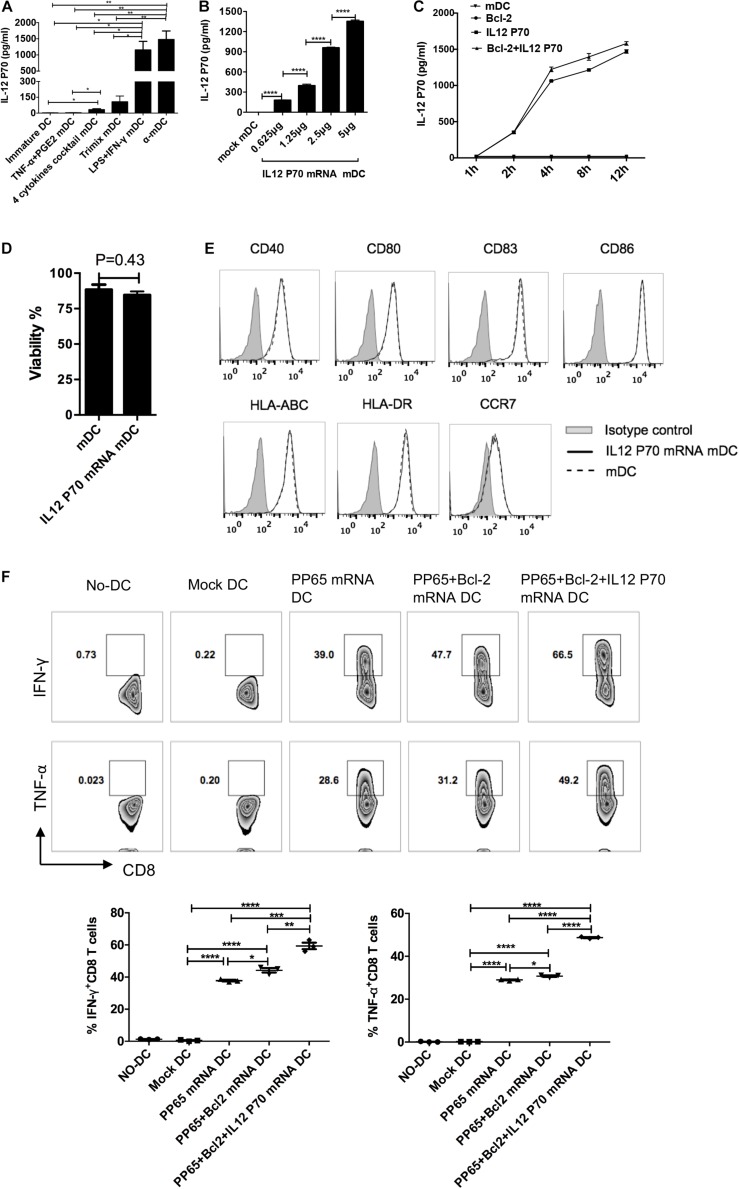
Exogenous IL-12p70 and Bcl-2 expression in human DCs provides strong CD8^+^ T cell priming capability. **(A)** Secretion of IL-12p70 by DCs matured with five clinical protocols. DCs were matured for 24 h, and IL-12p70 in supernatants was measured by ELISA. **(B)** IL-12p70 production by DCs transfected with different amounts of mRNAs (0.625, 1.25, 2.5, and 5 μg IL-12p70 mRNA per 10^6^ DCs) at 24 h. **(C)** Time course of IL-12p70 production in the supernatants after mRNA electroporation. **(D)** Effect of IL-12p70 mRNA electroporation on cell survival. **(E)** Effect of exogenous IL-12p70 expression on DC maturation status. **(F)** Priming capability of mDCs with the indicated modifications. DCs matured with the four-cytokine cocktail protocol were electroporated with pp65, pp65 + Bcl-2, or pp65 + Bcl-2 + IL-12p70 mRNAs (5 μg mRNA/10^6^ DCs), respectively. Two hours after electroporation, the mRNA-loaded-mDCs were co-cultured with non-adherent cells. The non-adherent cells were re-stimulated on day 7. On day 14, antigen-specific CD8^+^ T cell responses (IFN-γ and TNF-α production) were assessed. Results of one of three independent experiments are shown (*n* = 3). ^∗^*p* ≤ 0.05, ^∗∗^*p* ≤ 0.01, ^∗∗∗^*p* ≤ 0.001, ^****^p ≤ 0.0001.

Secretion of IL-12p70 by DCs is crucial for inducing cytotoxic T cell responses (Curtsinger et al., 1999). We first compared IL-12p70 secretion by mDCs prepared with the above five protocols. The mDCs stimulated by the α-DC-cytokine cocktail and LPS plus IFN-γ protocols secreted high levels of IL-12p70 (Figure 5A). By contrast, mDCs matured with TNF-α plus PGE2 or the four-cytokine cocktail secreted low levels of IL-12p70, possibly owing to inhibition by PGE2 (Jonuleit et al., 1997; Kalinski et al., 1997; van der Pouw Kraan et al., 1995). To exogenously express IL-12p70 in mDCs, we transfected different amounts of IL-12p70 mRNA into DCs matured with the four-cytokine cocktail protocol. IL-12p70 in the supernatant 24 h post electroporation was measured by ELISA. The amount of IL-12p70 in the supernatants was directly correlated with the amount of electroporated IL-12p70 mRNA (Figure 5B). We then analyzed the expression kinetics of the exogenous IL-12p70 after mRNA electroporation (5 μg mRNA/10^6^ DCs). A significant increase in IL-12p70 secretion was observed 2–4 h after electroporation, and the rate of IL-12p70 production slowed with time (Figure 5C). We further determined the effect of exogenous IL-12p70 mRNA expression on T cell priming. Exogenous IL-12p70 expression into DCs did not affect cell survival (Figure 5D) or maturation status (Figure 5E). We transfected DCs with pp65, pp65 + Bcl-2, and pp65 + Bcl-2 + IL-12p70 mRNAs and induced their maturation with the four-cytokine cocktail protocol. As shown in Figure 5F, DCs expressing exogenous Bcl-2 and IL-12p70 exhibited the strongest capability for priming CMV-specific CD8^+^ T cells. The frequencies of IFN-γ^+^ CD8^+^ T cells stimulated with pp65, pp65 + Bcl-2, and pp65 + Bcl-2 + IL-12p70 mRNA-loaded DCs were 37.8 ± 0.9%, 44.2 ± 2.3%, and 59.4 ± 3.6%, respectively, and the frequencies of TNF-α^+^CD8^+^ T cells were 29.0 ± 0.3%, 30.7 ± 0.6%, and 48.7 ± 0.2%, respectively. Taken together, these results indicate that exogenous IL-12p70 and Bcl-2 expression synergistically promote the ability of human DCs to prime CD8^+^ T cell responses.

## Discussion

As professional antigen-presenting cells, DCs loaded with tumor antigens have been used in clinical trials for cancer immunotherapy (Constantino et al., 2016). Owing to the limited availability of primary human DCs, various stimulation strategies are used to differentiate monocytes into immature DCs and then mDCs. In this study, we aimed to enhance the current protocols for rapid clinical applications. We first compared the current five clinical protocols and determined that the four-cytokine cocktail protocol was the preferred method to be used for further modification. Our modified protocol endows DCs with the following four features: (a) fully mature status with high expression of co-stimulatory molecules and HLA molecules; (b) upregulated CCR7 expression for high migration capacity toward lymph nodes; (c) improved cell survival through exogenous Bcl-2 expression; (d) secretion of sufficient levels of IL-12p70 through exogenously expressed IL-12p70. The resulting mDCs with these features are potent antigen-presenting cells and can induce strong CD8^+^ T cell responses *in vitro*. Therefore, this modified protocol represents a promising new means to produce potent human DCs for cancer immunotherapy.

The maturation status of DCs is crucial to their induction of T cell responses (Zhou and Tedder, 1996; Sabado and Bhardwaj, 2010). The four-cytokine cocktail protocol induced a stronger overall maturation phenotype of mDCs than the other four protocols. Although the α-DC-cytokine cocktail protocol also induced high levels of CD40 and CD86 on mDCs, expression of CD83, HLA-ABC, and HLA-DR was significantly lower compared with that of mDCs matured by the four-cytokine cocktail protocol. In addition, the ability of DCs to migrate to lymph nodes is critical for inducing immune responses, and improved DC vaccine migration could improve overall survival of patients (Sabado et al., 2017). Although α-DC induced more peptide-specific T cells than the DCs matured by the four-cytokine cocktail *in vitro*, the low expression level of CCR7 on α-DC limited its clinical application. Moreover, it has been reported that mDCs matured with the α-DC-cytokine protocol cannot efficiently express exogenous mRNA genes (Bontkes et al., 2007). However, the four-cytokine cocktail and TNF-α plus PGE2 protocols induced high levels of CCR7 expression on DCs, consistent with other studies (Kabashima et al., 2003; Legler et al., 2006; Scandella et al., 2002). Thus, we chose the four-cytokine cocktail protocol as the base protocol for further improvement.

Previous studies have shown that DCs are prone to cell death during migration to lymphoid organs (Fossum, 1988; Pugh et al., 1983), and fewer than 5% of infected DCs reach the lymph nodes (De Vries et al., 2003). Furthermore, DC maturation was accompanied by increased cell death (Spisek et al., 2001). Our comparison revealed that mDCs induced by three protocols (the four-cytokine cocktail, the α-DC-cytokine cocktail, and TNF-α plus PGE2) retained high levels of cell viability 24 h post maturation. However, viability of mDCs rapidly decreased to <20% by day 5 in the absence of stimulation factors, mimicking the *in vivo* situation (Figure 4C). Indeed, the viability of DCs administered as vaccines rapidly decreases at the injection site after intranodal or intradermal delivery (Verdijk et al., 2009). We reasoned that enhancing mDC survival would increase T cell priming. Bcl-2 is a critical pro-survival factor that is rapidly downregulated during DC maturation (Granucci et al., 2001a, b; Nopora and Brocker, 2002). Bcl-2-transgenic DCs induce T cell activation more efficiently than wild-type DCs in mice (Nopora and Brocker, 2002). Administration of DNA encoding Bcl-2 or Bcl-xL has been reported to improve DNA vaccine potency (Pirtskhalaishvili et al., 2000; Kim et al., 2003). Similar approaches have been reported to improve the survival of DCs through silencing of the pro-apoptotic genes Bim (Kim et al., 2009) and Bak/Bax (Kang et al., 2007). As mRNA does not interact with the genome, an mRNA-based vaccine offers safety advantages. To alleviate the oncogenicity risk of Bcl-2, Bcl-2 mRNA was electroporated into mDCs. Indeed, Bcl-2 mRNA electroporation led to a more than twofold increase in the viability of DCs compared with controls. Furthermore, introducing Bcl-2 into human mDCs resulted in stronger antigen-specific CD8^+^ T cell responses *in vitro*. These data suggest that Bcl-2 mRNA electroporation could be used to improve DC vaccine efficacy by increasing the viability of DCs.

IL-12p70 secretion can enhance the ability of DCs to induce T cell responses (Mailliard et al., 2004; Park et al., 2011; Lee et al., 2008), and higher levels of IL-12p70 production were associated with the clinical benefit of DC vaccines (Okada et al., 2011; Hansen et al., 2012; Carreno et al., 2013). As PGE2 in the four-cytokine cocktail protocol suppresses IL-12p70 production (Jonuleit et al., 1997; Kalinski et al., 1997; van der Pouw Kraan et al., 1995), exogenous expression of IL-12p70 mRNA in DCs matured by the four-cytokine cocktail protocol was necessary. Our results showed that exogenous IL-12p70 expression in mDCs did not affect their survival and maturation and, together with Bcl-2, dramatically enhanced DC priming of antigen-specific CD8^+^ T cell responses. Notably, mDCs start to secrete IL-12p70 2 h after electroporation, with a significant increase at 2–4 h, after which the rate of IL-12p70 production slows down. These expression kinetics suggest that IL-12p70 mRNA-loaded DCs should be inoculated into patients within 2–3 h after electroporation. Alternatively, the mRNA should be modified to increase protein expression levels and prolong IL-12 secretion (Mockey et al., 2006; Bontkes et al., 2007). It has been reported that most of the cytokines are produced within 24 h after DC maturation (Dohnal et al., 2009). Therefore, exogenous expression of IL-12p70 mRNA may be preferred for clinical use.

In conclusion, mDCs transfected with Bcl-2 and IL-12p70 mRNAs and matured by the four-cytokine cocktail protocol express high levels of costimulatory and HLA molecules, show enhanced cell survival, and produce IL-12p70. These DCs can prime a strong antigen-specific CD8^+^ T cell response. Our results suggest that the modified protocol is suitable for clinical applications.

## Data Availability Statement

All datasets generated for this study are included in the article/supplementary material.

## Ethics Statement

Ethical review and approval was not required for the study on human participants in accordance with the local legislation and institutional requirements. Written informed consent for participation was not required for this study in accordance with the national legislation and the institutional requirements.

## Author Contributions

Y-WH and HS designed the study. HZ and YW carried out the experiments. Q-TW, S-NS, and S-YL helped perform the data analysis. HZ and Y-WH wrote the manuscript. All authors read and approved the final manuscript.

## Conflict of Interest

Y-WH and S-YL are founders and shareholders of Tricision Biotherapeutics Inc. Tricision Biotherapeutics has no role in the designing and executing the experiments. The remaining authors declare that the research was conducted in the absence of any commercial or financial relationships that could be construed as a potential conflict of interest.

## Abbreviations

caTLR4: constitutively active Toll-like receptor 4
CMV: cytomegalovirus
DC: dendritic cells
GM-CSF: granulocyte-macrophage colony-stimulating factor
IL-12: interleukin-12
mDC: mature DC
PBMC: peripheral blood mononuclear cell.
